# Molecular Characterization of a Novel *N*-Acetylneuraminate Lyase from a Deep-Sea Symbiotic Mycoplasma

**DOI:** 10.3390/md16030080

**Published:** 2018-03-05

**Authors:** Shao-lu Wang, Yun-liang Li, Zhuang Han, Xi Chen, Qi-jia Chen, Yong Wang, Li-sheng He

**Affiliations:** 1 Department of Life Sciences, Institute of Deep-sea Science and Engineering, Chinese Academy of Sciences, Sanya 572000, China; wangsl@idsse.ac.cn (S.-l.W.); zhuanghan@idsse.ac.cn (Z.H.); wangy@idsse.ac.cn (W.Y.); 2 College of Earth Sciences, University of Chinese Academy of Sciences, Beijing 100864, China; 3 Laboratory of Soft Matter Physics, Institute of Physics, Chinese Academy of Sciences, Beijing 100864, China; yunliangli@iphy.ac.cn; 4 National Engineering Laboratory for Industrial Enzymes, Tianjin Institute of Industrial Biotechnology, Chinese Academy of Sciences, Tianjin 300308, China; chen_x@tib.cas.cn (X.C.); chen_qj@tib.cas.cn (Q.-j.C.)

**Keywords:** *N*-acetylneuraminate lyase, Symbiotic microbe, *N*-acetylneuraminic acid, mycoplasma

## Abstract

*N*-acetylneuraminic acid (Neu5Ac) based novel pharmaceutical agents and diagnostic reagents are highly required in medical fields. However, *N*-acetylneuraminate lyase（NAL）for Neu5Ac synthesis is not applicable for industry due to its low catalytic efficiency. In this study, we biochemically characterized a deep-sea NAL enzyme (abbreviated form: MyNal) from a symbiotic Mycoplasma inhabiting the stomach of a deep-sea isopod, *Bathynomus jamesi*. Enzyme kinetic studies of MyNal showed that it exhibited a very low K_m_ for both cleavage and synthesis activities compared to previously described NALs. Though it favors the cleavage process, MyNal out-competes the known NALs with respect to the efficiency of Neu5Ac synthesis and exhibits the highest *k_cat_*/K_m_ values. High expression levels of recombinant MyNal could be achieved (9.56 mol L^−1^ culture) with a stable activity in a wide pH (5.0–9.0) and temperature (40–60 °C) range. All these features indicated that the deep-sea NAL has potential in the industrial production of Neu5Ac. Furthermore, we found that the amino acid 189 of MyNal (equivalent to Phe190 in *Escherichia coli* NAL), located in the sugar-binding domain, GX189DE, was also involved in conferring its enzymatic features. Therefore, the results of this study improved our understanding of the NALs from different environments and provided a model for protein engineering of NAL for biosynthesis of Neu5Ac.

## 1. Introduction

**Figure 1:**
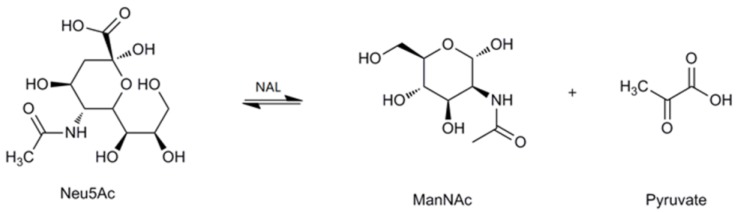
The reversible reaction catalyzed by *N*-acetylneuraminate lyase (NAL).

Sialic acids belong to a family of nine-carbon amino sugars. They are abundantly present as the outermost components of complex mucoid substances, such as glycoproteins and glycolipids [1,2,3]. Sialic acids are ubiquitous in nature and participate in a variety of biological, pathological and immunological processes, such as molecular recognition, cell-cell communication, bacterial and viral infection, and tumor metastasis [4,5,6]. To date, more than 50 distinct sialic acid structures have been recognized; among these, the most abundant and extensively studied sialic acid is *N*-acetylneuraminic acid (Neu5Ac) [7,8]. Due to its multiple roles, especially in neurogenesis and anti-microbial infection, Neu5Ac has been used commercially as an anti-influenza drug and as an important additive in dairy products [9,10]. Recently, novel sugar-chain-related pharmaceutical compounds such as therapeutic agents and diagnostic reagents for infectious diseases, autoimmune diseases, cancer and nerve diseases are being developed on the basis of the multi-functionality of Neu5Ac. As a result, therapeutic agents for influenza have recently been realized. A new era has thus been opened for the practical application of Neu5Ac in the medical field [9,11]. In the past, Neu5Ac has been extracted from natural sources, such as colominic acid, milk, and egg yolk; however, at present, Neu5Ac can also be synthesized by chemical or enzymatic methods [12,13,14,15]. The enzyme used for the biosynthesis of Neu5Ac is *N*-acetylneuraminate lyase (NAL, EC 4.1.3.3). It is also called sialic acid aldolase, and it catalyzes the reversible aldol cleavage of Neu5Ac to generate *N*-acetyl-d-mannosamine (ManNAc) and pyruvate (Figure 1).

Reversibly, NAL might produce Neu5Ac when the available substrate levels favor Neu5Ac synthesis. Nowadays, NAL is extensively used in the synthesis of sialic acids and its analogues, though the efficiency is not high during the industrial production of Neu5Ac [13,16,17]. Since NALs are economically important for sialic acid synthesis, various NALs have been obtained from animals as well as numerous bacterial species [18,19,20]. NALs have previously been cloned from *Escherichia coli*, *Clostridium perfringens*, *Pasteurella multocida*, *Haemophilus influenzae*, and *Trichomonas vaginalis* [21,22,23,24,25]. In animals, the primary function of the enzyme is to regulate intracellular sialic acid metabolism. However, in microorganisms, NALs enable the bacteria to utilize sialic acids as a source of organic carbon in addition to the regulatory functions of NALs. In contrast, in certain *E. coli* strains, the enzyme has been reported to act in the reverse direction under some circumstances, thereby, enabling the biosynthesis of sialic acids [26,27,28,29].

NAL from *E. coli*, *C. perfringens*, *Lactobacillus plantarum*, *Staphylococcus carnosus*, and *Corynebacterium glutamicum* have been characterized in detail [30,31,32,33,34]. Subsequently, the enzymes from *E. coli* and *H. influenzae* have been crystallized for analyzing their X-ray structures [24,35,36,37]. However, NAL candidates from the deep sea and other extreme environments have not been studied yet. Due to the inadequacy of nutrients in deep-sea zones, the enzymes expressed by deep-sea organisms for carbon degradation are extremely efficient and have some special enzymatic features. So far, some novel enzymes identified from the deep sea and other extreme environments are found to be associated with special activities and tolerance, as exemplified by proteases, lipases, and esterases [38,39,40]. Recently, we discovered a novel NAL in a symbiotic Mycoplasma within the stomach of a deep-sea isopod [41]. In this study, we conducted molecular investigations on the NAL from symbiont *Mycoplasma* sp.(MyNal) and compared its biosynthesis efficiency and environmental tolerance with that of other known NALs. 

## 2. Results

### 2.1. Cloning, Expression, and Purification of MyNal

The MyNal gene was obtained from a *Mycoplasma* sp. symbiont with 885 base-pairs (GenBank No. MG188686). The recombinant MyNal was expressed in *E. coli* as a fusion protein with an approximate molecular weight of 52 kDa, as shown in Figure S1. Almost all of the expressed MyNal was in a soluble form under optimal conditions and reached a peak level of 9.56 mol in 1 L culture (Table S1).

### 2.2. Biochemical Characterization of Recombinant MyNal

**Figure 2:**
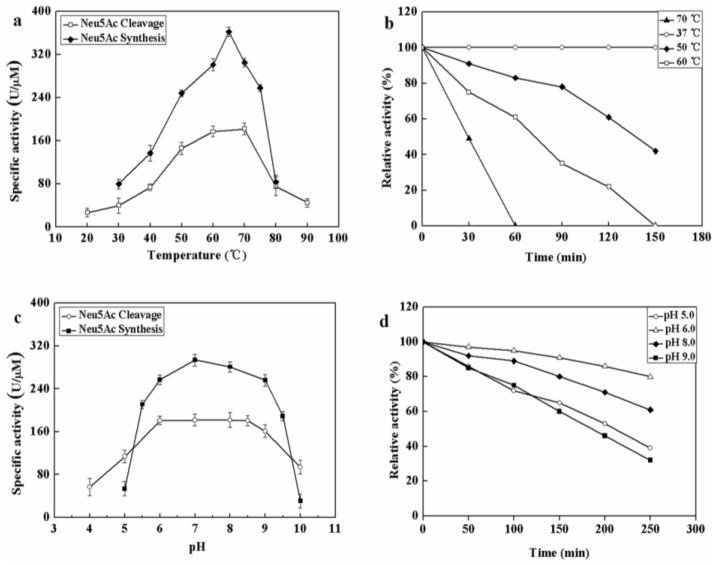
Effect of pH and temperature on MyNal activity and stability. (**a**) Temperature profile for Neu5Ac hydrolysis and synthesis. Synthetic assays were performed with 30 mM ManNAc, 100 mM pyruvate and 38.5 × 10^−5^ μM of enzyme at pH 7.0; hydrolytic assays were performed with 4 mM Neu5Ac and 2.9 × 10^−5^ μM of enzyme at pH 7.0. (**b**) Temperature-stability of MyNal in the hydrolysis direction. MyNal was incubated for different periods of time at 37 °C, 50 °C, 60 °C and 70 °C, and its activity was measured by spectrophotometry under the standard reaction conditions at pH 7.0, using Neu5Ac as the substrate. (**c**) pH profile for Neu5Ac hydrolysis and synthesis. The contents of the reaction mixture were the same as described above; the reactions were performed at optimum temperature (the optimum temperature for cleavage was 70 °C, while for synthesis was 60 °C) at different pHs. The pH value was adjusted by using 20 mM acetic acid (pH 4.0–6.5), phosphate buffer (pH 7.0–7.5), tris-HCl (pH 8.0–9.5), or glycine-NaOH (pH 10.0–11.0). (**d**) pH-stability of MyNal during hydrolysis. MyNal was incubated for different periods of time at pH values of 5.0, 6.0, 8.0, and 9.0, and the activity was measured spectrophotometrically at 37 °C using Neu5Ac as the substrate.

The effect of temperatures on MyNal activity was examined for both Neu5Ac cleavage and synthesis. Results showed that the optimal temperature for cleavage activity was 70 °C in the test range. When the incubation temperature was decreased to 50 °C, 80% of the cleavage activity was still retained. The sharp decline in the activity at temperatures <50 °C or >70 °C indicates that the performance of MyNal might be optimal at temperatures of 50 °C–70 °C. This revealed that the optimal temperature range for MyNal was much wider than that reported in previous studies of NALs (Figure 2a). The optimal temperature for synthetic activity of MyNal was 65 °C, which was higher than that previously described for CgNal (40 °C) [34], ScNal (50 °C) [33], and LpNal (60 °C) [32]. The activity of MyNal decreased sharply below or above 65 °C. To test its thermal stability, MyNal was maintained at different temperatures for 150 min. Results showed that MyNal was quite stable at 37 °C and remained stable after incubation for 150 min. The stability decreased with an increase in the incubation temperature. When incubated at 70 °C, the half-life for enzymatic activity was 30 min and that for a complete loss of activity was 1 h (Figure 2b). To investigate the effect of pH on MyNal activity, its activities during cleavage and synthesis were examined in the pH range of 4.0–10.0. Results showed that the activities of MyNal for performing the cleavage and synthesis reactions were similarly affected by variations of pH between 6.0 and 8.5, and were the highest at pH 7.0 (Figure 2c). In contrast, other NALs did not show a plateau of pH tolerance, except during the synthesis activity of LpNAL, which was performed stably in the pH range of 6.0–10.0 [32]. The pH-stability of MyNal for cleavage was determined by incubating the enzyme at different pHs for more than four hours. The enzyme was observed to be stable under acid (pH 6.0) and alkaline (pH 8.0) conditions, where it maintained around 95% residual cleavage activity after 120 min incubation at pH 6.0, and 85% residual cleavage activity after 120 min incubation at pH 8.0 (Figure 2d).

**Figure 3:**
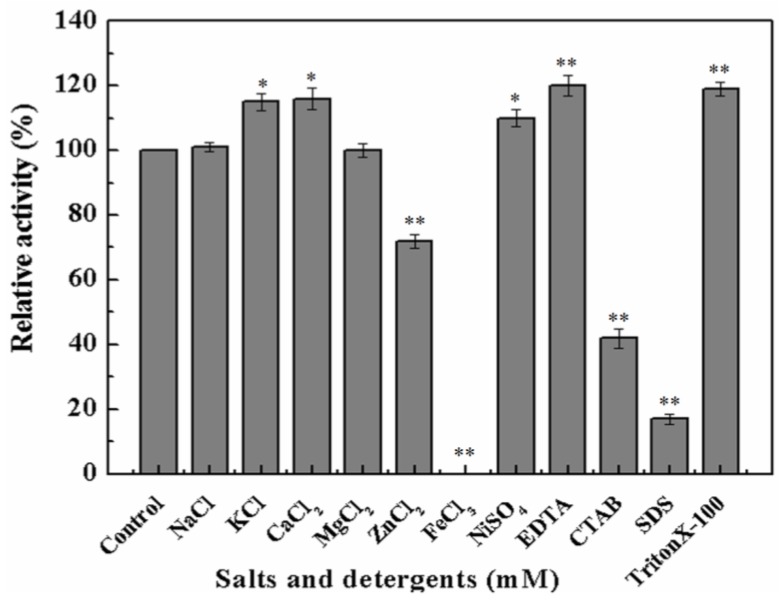
Effects of salts and detergents on MyNal activity during cleavage. Enzyme activity was spectrophotometrically assayed in standard reactions after incubation of purified MyNal (2.9 × 10^−5^ μM) with different compounds (5 mM), as indicated in the figures, for 15 min at 37 °C; enzymatic activities were considered to be 100% when salts and detergents were excluded. Statistical significance was calculated for treated samples compared to the control. * *p* < 0.05 ; ** *p* < 0.01.

To further investigate whether metal ions or chemical inhibitors affected MyNal cleavage activity, various metals and chemicals were tested. Results showed that MyNal activity was stabilized or significantly stimulated in the presence of Na^+^, K^+^, Ca^2+^, Mg^2+^, and Ni^2+^ (Figure 3). In comparison, Fe^3+^ imposed a strong inhibitory effect, and 5 mM FeCl_3_ might fully inactivate MyNal activity. MyNal cleavage activity was also significantly affected by 5 mM ZnCl_2_, CTAB, and SDS, with reductions of 30%, 40%, and 20%, respectively. On the other hand, the cleavage activity was significantly enhanced by EDTA and TritonX-100 at test concentrations (Figure 3).

**Figure 4:**
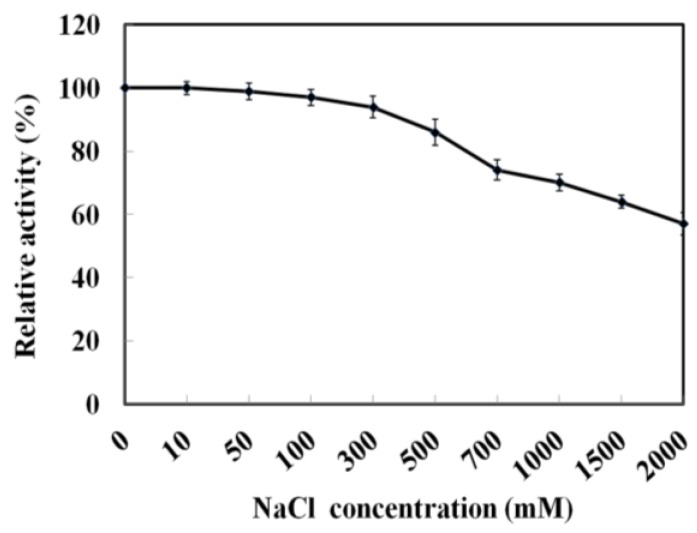
Effect of salinity on MyNal activity during cleavage. The enzymatic activity was spectrophotometrically assayed in the standard reaction after incubation with purified MyNal (2.9 × 10^−5^ μM) at different NaCl concentrations. The enzyme activity was considered to be 100% when NaCl was excluded from the reaction mixture.

As MyNal was cloned from a marine organism, it is perhaps more tolerant to a high salt concentration. The assay was performed with salt concentrations in the range of 0–2 M. As shown in Figure 4, MyNal cleavage activity was not influenced by salt and showed a relative stable activity when salt concentrations were <300 mM. When the salt concentration was above 300 mM, a slow decline in the activity was observed. When the concentration was increased to 2 M, the activity was still maintained at 60%.

### 2.3. Kinetic Parameters of MyNal

The kinetic constants for Neu5Ac cleavage and synthesis were determined at an optimal temperature and pH, as shown in Table 1. The K_m_ value for Neu5Ac cleavage was 1.8 ± 0.2 mM, similar to that of LpNAL [32], but was much lower than that reported for other NALs. For the synthesis process, the K_m_ values were 6.9 ± 0.5 mM and 5.9 ± 0.9 mM for ManNAc and pyruvate, respectively, which were also much lower than those for CgNal [34], and were the lowest reported K_m_ value to date. The *k_cat_*/K_m_ values for ManNAc and pyruvate were 1.86 s^−1^mM^−1^ and 1.12 s^−1^mM^−1^, respectively, and were the highest *k_cat_*/K_m_ value to date. Although MyNal had a lower catalytic efficiency, the synthesis activity of MyNal was 16.9- and 2.73-fold higher than that of CgNal (Table 1).

### 2.4. Mutagenetic Analyses of Recombinant MyNal

**Figure 5:**
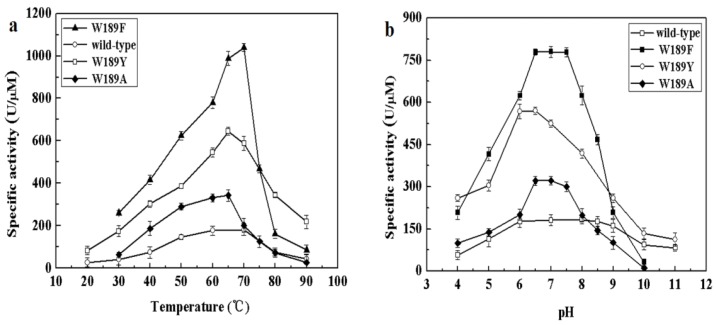
Effect of temperature and pH on the activity of mutants. (**a**) Temperature profile for Neu5Ac hydrolysis. Assays were performed with 4 mM Neu5Ac and 2.9 × 10^−5^ μM of enzyme at pH 7.0. (**b**) pH profile for Neu5Ac hydrolysis. The contents of the reaction mixture were the same as described above; the reactions were performed at optimum temperature with different pHs. The pH value was adjusted by using 20 mM acetic acid (pH 4.0–6.5), phosphate buffer (pH 7.0–7.5), tris-HCl (pH 8.0–9.5), or glycine-NaOH (pH 10.0–11.0).

The different composition of amino acids making up the NALs may be accountable for the above differences in cleavage and synthesis efficiency under different conditions. We therefore examined the alignment of NALs and identified differences in somewhat conserved sites. Amino acid 189 of MyNal (equivalent to Phe190 in EcNal) in the sugar binding domain GW189DE differs from that of other NALs (Figure S2). Although the amino acid in the 189 position was not highly conserved, we were interested in investigating whether W189 was attributable to the outstanding performance of MyNal. The optimal temperature, pH, and kinetic parameters were tested among three mutants; the three mutants shared similar optimal temperatures and pHs with the MyNal wild-type (Figure 5). However, the three mutants had a much lower K_m_ than the wild-type, resulting in W189Y and W189F having K_m_ values that were one-third and one-fifth, respectively, of the K_m_ value for the wild-type for the cleavage direction. In addition, the *k_cat_*/K_m_ values of W189A, W189Y, and W189F were 3- to 10-fold higher than those of the wild-type (Table 2).

### 2.5. Effect of Pressure on Activity of MyNal

**Figure 6:**
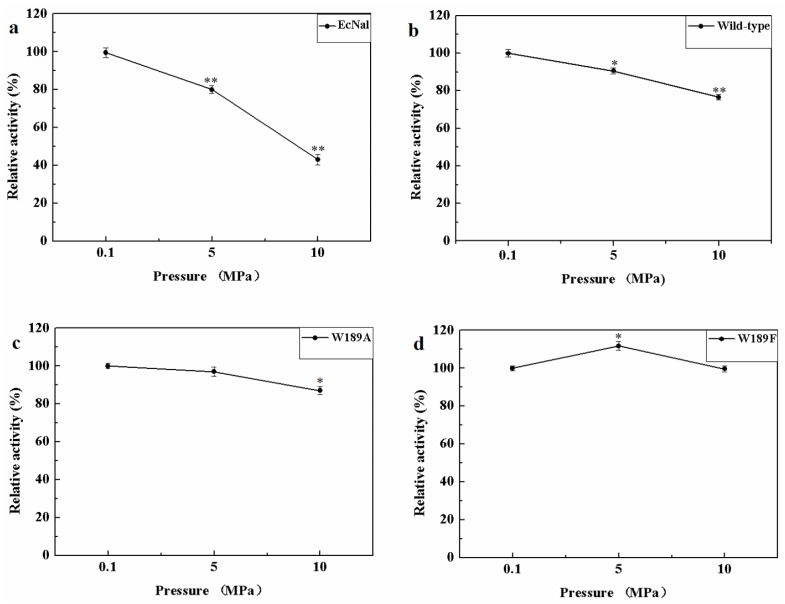
Effects of pressure on enzyme activity during cleavage. (**a**) Pressure profile for EcNal. Hydrolytic assays contained 4 mM Neu5Ac and 2.9 × 10^−5^ μM of enzyme were performed at different pressures (atmospheric pressure (0.1 MPa), 5 MPa, 10 MPa) and room temperatures (RT). (**b**) Pressure profile for wild-type enzyme MyNal. Assays were performed the same as above. (**c**) Pressure profile for mutant W189A. Assays were performed the same as above. (**d**) Pressure profile for mutant W189F. Assays were performed the same as above. Statistical analyses were performed on the higher pressure treated samples. * *p* < 0.05; ** *p* < 0.01.

MyNal was isolated from a deep-sea animal captured at a depth of 1000 m, and thus, it is debatable whether MyNal is pressure sensitive. Three sets of pressure tolerant assays were conducted at room temperature. As shown in Figure 6, the relative activity was roughly negatively correlated with the increase in pressure for EcNal. EcNal cleavage activity was reduced to 80% and 40% under 5 MPa and 10 MPa, respectively, compared to the cleavage activity observed at 0.1MPa (Figure 6a). For MyNal, the cleavage activity was measured as 90% of its original activity at 5 MPa and 80% of the activity at 10 MPa (Figure 6b). The MyNal mutants showed a similar result (Figure 6c,d).

## 3. Discussion

Marine microorganisms are novel enzyme sources. However, due to difficulties related to the sampling process, most of these enzymes have not been isolated and examined [43]. Their biological importance and key role in pathogenic infections of sialic acids have rendered the enzymatic studies of *N*-acetylneuraminate lyase a subject of great interest. To date, there have been several reports of *N*-acetylneuraminate lyase from terrestrial organisms, but there are not many studies on those from marine microorganisms. Although the potential to acquire great knowledge is associated with abundant resources for research and development, only a few studies have provided knowledge about the biochemical characteristics of NAL enzymes. In the present study, we investigated a novel *N*-acetylneuraminate lyase, MyNal, from deep-sea symbiotic Mycoplasmas, to understand its enzymatic kinetics as well as the varied parameters impacting its enzyme kinetics.

In this study, MyNal displayed a very low K_m_ for both cleavage and synthesis activities, indicating that it had a greater affinity to the substrates Neu5Ac, ManNAc, and pyruvate than other NALs. In addition, this enzyme had a high *k_cat_*/K_m_ value that was more than ten-fold higher than that of the highest *k_cat_*/K_m_ value previously reported for CgNal (Table 1), especially for synthesizing Neu5Ac using ManNAc and pyruvate as substrates. Therefore, MyNal is the most efficient enzyme for Neu5Ac synthesis. Site 189 of MyNal in the sugar-binding domain GXDE differs from that of other NALs, as shown in Figure S2. Most enzymes of the NAL subfamily adopt 189F or 189Y in the NAL group, while MyNal adopts 189W. Previous reports have shown that the amino acid G before 189W in the GWDE motif and the two amino acids (D and E) after 189W were highly conserved and considered to be critical for NAL activity [42,44]. Although the amino acid at the 189 location was not conserved in MyNal, we were interested in investigating whether W189 influenced MyNal activity. In order to clarify the functional role of the non-conserved site189, W189A, W189Y, and W189F variants of MyNal were constructed. The three mutants of MyNal exhibited a stronger catalytic activity than that of the wild-type. Hence, enzymatic activity was not inhibited at this site in the mutants, but the efficiency of the enzyme was found to be changed. This provides insights into the bioengineering of new NALs for creating the desired enzymatic structure.

In the current study, MyNal showed slightly higher salt-tolerance ability up to 2M, maybe because of its previous growth in a salty marine environment. To date, there has been no report on the salt-tolerance of other NALs. It is difficult to state whether the salt-tolerance trait was specific to MyNal. At present, the activity of a few enzymes has been studied under pressure. The activity of lactate dehydrogenase (LDH) has been previously studied for both deep-sea and coastal fishes, which revealed that recombinant LDH from deep-sea species exhibited a significantly higher tolerance to pressure than coastal fish [45,46]. In our study, MyNal and its mutants could tolerate a hydrostatic pressure of up to 10 MPa, suggesting that NAL from Mycoplasmas evolved to develop a strong capacity for pressure tolerance. Dihydrofolate reductase is one of the most extensively studied enzymes for structural specificities coupled to pressure tolerance. A previous study has shown the close relationship between cavity formation, hydration, and pressure adaptation [47]. Because cavity formation and hydration depend on amino acid side chains, enzymes could adapt to the deep-sea environment by substituting the side chains with other amino acid groups without changing their backbone structure [48]. Aside from the shared conserved catalytic domains, MyNal and EcNal differ significantly and are only 35% identical at the amino acid level. We propose that the non-conserved amino acids of MyNal probably contribute to protein cavity formation and hydration, which are critical for environmental adaptation [49].

The *N*-acetylneuraminate lyase from symbiotic Mycoplasmas has special characteristics compared to other reported NALs and provides new information for biological engineering of NAL for desired applications. 

## 4. Materials and Methods

### 4.1. Strains, Plasmids and Chemicals

Symbiotic Mycoplasma genomic DNA was obtained from the stomach contents and stomach sac of the deep-sea isopod *Bathynomus jamesi*. The deep-sea isopod *Bathynomus jamesi* was captured at a depth of 1000 m using a trap set on a lander during a cruise in the South China Sea (110°38′13.02″E, 17°46′50.7″N). *E. coli* DH5α and BL21(DE3) cells were obtained from Takara (Tokyo, Japan). The pET-32a(+) vector was obtained from Novagen (Madison, WI, USA), while restriction enzymes, T4-DNA ligase, DNA markers, and protein markers were obtained from Thermo Scientific (Waltham, MA, USA). The QIAquick PCR purification kit was obtained from Qiagen (Venlo, The Netherlands). *N*-acetyl-d-mannosamine and *N*-acetylneuraminic acid were obtained from Sigma-Aldrich (Shanghai, China). All other reagents were of analytical grade and are commercially available from Sigma-Aldrich (Shanghai, China).

### 4.2. Cloning and Mutagenesis

The MyNal gene was amplified by PCR using the genomic DNA of the symbiotic Mycoplasma as a template. There is a stop codon (TGA) in the MyNal sequence, which was translated into tryptophan in Mycoplasma. The mutagenesis of TGA and later W189A, W189Y, and W189F variants were generated by using the full-length MyNal-plasmid as a template. The purified and digested PCR product was ligated with a pET-32a(+) vector. The positive clonings were identified by sequencing and then expressed in BL21 (DE3) competent cells. The primers used in this study have been listed in Table S2.

### 4.3. Expression and Purification of MyNal

*E.coli* cells harboring the recombinant plasmid pET32a-MyNal were cultured overnight at 37 °C in 10 mL of LB medium containing 100 μg/mL ampicillin, before being transferred to a 2-liter flask containing 500 mL of LB broth with the above-mentioned antibiotic. The entire overnight-incubated culture was inoculated into 500 mL of prewarmed media and grown for another 2–3 h at 37 °C with vigorous shaking until an OD_600_ of ~0.8 was reached. The expression of MyNal or its variants was induced using a final concentration of 0.5 mM Isopropyl-β-d-thiogalactoside (IPTG), and then incubated for an additional 4–6 h at 30 °C. Cells were harvested and then resuspended in 10 mL of lysis buffer (50 mM NaH_2_PO_4_, 300 mM NaCl, 10 mM imidazole, pH 8.0). After ultrasonication, the cell debris was removed by centrifugation and the supernatant was retained. The enzyme was purified directly from the lysate using a Ni^2+^-NTA affinity column. The supernatant was applied to a 1-mL Ni-NTA Agarose column, which was pre-equilibrated in the native lysis buffer and then washed with a linear gradient of imidazole in wash buffer (50 mM NaH_2_PO_4_, 300 mM NaCl, 20–80 mM imidazole, pH 8.0). Then, the target proteins were eluted with 5 mL of native elution buffer (50 mM NaH_2_PO_4_, 300 mM NaCl, 350 mM imidazole, pH 8.0). Fractions exhibiting MyNal activity were pooled and dialyzed at 4 °C for 24 h against three changes of 1X PBS and then stored at −80 °C for further experiments. All of the purification procedures were performed on ice. The homogeneity of MyNal and the molecular mass of its subunit were determined by 12% sodium dodecyl sulfate polyacrylamide gel electrophoresis (SDS-PAGE). The concentration of purified enzymes was determined using Bradford’s reagent (BioRad, Hercules, CA, USA), with bovine serum albumin used as a protein standard.

### 4.4. Enzyme Assay of MyNal

MyNal activity was assayed by measuring its ability to cleave Neu5Ac into ManNAc and pyruvate (Neu5Ac cleavage), as well as its ability to condense ManNAc and pyruvate into Neu5Ac (Neu5Ac synthesis). MyNal cleavage activity was determined by measuring the amount of pyruvate, one of the cleavage products using lactate dehydrogenase (LDH) and the reduced form of NADH, which can be spectrophotometrically quantified at 340 nm [50]. The standard reaction was performed in 1mL medium containing 20 mM phosphate buffer (pH 7.0), 150 μM NADH, 0.5 U LDH, 4 mM Neu5Ac, and 2.9 × 10^−5^ μM of purified MyNal. MyNal synthetic activity was determined from the increase in the Neu5Ac peak area using high performance liquid chromatography (HPLC) [34]. Samples were analyzed on an Agilent 1100 system equipped with a Bio-Rad Aminex HPX-87H column (300 mm × 7.8 mm, 9 μm), and a mobile phase consisting of 5 mM H_2_SO_4_ running at 0.4 mL/min at 40 °C. Under these conditions, the retention times for Neu5Ac, ManNAc, and pyruvate were 11.9 min, 16.2 min, and 14.2 min, respectively (Figure S3). The standard reaction contained 30 mM ManNAc, 100 mM pyruvate, and 38.5 × 10^−5^ μM of purified MyNal in 20 mM phosphate buffer (pH 7.0). All tests were performed at least three times. One enzymatic unit was defined as the number of micromoles of NADH hydrolyzed per minute per micromole of enzyme (spectrophotometer) or the amount of enzyme required to synthesize 1 μmol of Neu5Ac in 1 min within the above reaction conditions (HPLC).

### 4.5. Enzyme Characterization of MyNal

To examine the effect of temperature on enzyme activity, a reaction involving 4 mM Neu5Ac (cleavage) or 100 mM pyruvate and 30 mM ManNAc (synthesis) in 20 mM phosphate buffer (pH 7.0) was carried out at different temperatures (20–90 °C). The reaction mixtures were incubated for 15 min after the enzyme was added at different temperatures, as mentioned above, and then inactivated at 99 °C for 5 min. The thermostability of enzymes was assayed by measuring the residual activities after heat treatment. The examined enzyme was incubated in 20 mM phosphate buffer (pH 7.0) at different temperatures, including 37 °C, 50 °C, 60 °C, and 70 °C, for 150 min at each temperature. The residual activities were measured by the standard method, as described above.

The effect of pH on enzyme activity was examined at different pHs ranging from 4.0 to 11.0 for carrying out both cleavage and synthesis reactions. Acetic acid was used for adjusting the pH value to the range of 4.0–6.5; similarly, tris-HCl, potassium phosphate buffer, and glycine-NaOH were used for adjusting the pH to 8.0–9.5, 7.0–7.5, and 10–11, respectively. The pH stability was assayed by incubating the enzyme in buffers with different pH values (5.0, 6.0, 8.0 and 9.0) at 37 °C for 250 min. The buffer used was the same as described above. The residual activities were detected by the method as described above.

The effect of metal ions on the enzyme’s cleavage activity was examined by using NaCl, KCl, CaCl_2_, MgCl_2_, NiSO_4_, FeCl_3_, and ZnCl_2_ at final concentrations of 5 mM. The enzyme was incubated with each ion at 37 °C for 15 min in 20 mM tris-HCl (pH 7.0). Residual activities were measured by using the standard assay described above. The effect of detergents on enzyme activity was investigated using ethylenediaminetetraacetic acid (EDTA), sodium dodecyl sulfate (SDS, 0.14%), cetyltrimethylammonium ammonium bromide (CTAB), and triton X-100 (0.32%) at a final concentration of 5 mM. Enzyme activity was monitored by the method described above. The reaction mixture in which salts and detergents were not included was used as the control.

The effect of salinity on the enzyme’s cleavage activity was assayed in different gradients of NaCl (10 mM, 50 mM, 100 mM, 300 mM, 500 mM, 700 mM, 1000 mM, 1500 mM, and 2000 mM). The activities were detected by the standard method described above. The reaction mixture in which NaCl was not included was used as the control.

The effect of pressure on the enzyme’s cleavage activity was examined in a reaction system containing 2.9 × 10^−5^ μM of purified enzyme and 4 mM of Neu5Ac as a substrate in 20 mM phosphate buffer (pH 7.0). Reaction systems were incubated at room temperature and pressures (atmospheric pressure, 5 MPa and 10 MPa) for 15 min. Enzyme activity was detected by the standard assay.

Kinetic parameters of MyNal were assayed by measuring enzyme activities in the presence of various concentrations of substrates. For determining the enzyme’s cleavage activity, 2.9 × 10^−5^ μM of enzyme and varied concentrations (0, 0.25, 0.5, 1, 1.25, 1.5, 1.75, 2, 3, and 4 mM) of Neu5Ac were used. For determining the synthesis activity, 38.5 × 10^−5^ μM of enzyme, a fixed concentration of pyruvate (20 mM), and varied concentrations (0, 2, 5, 10, 15, 20, 25, 30, 35, 40, 45, 50, and 60 mM) of ManNAc were used. Alternatively, a fixed concentration of ManNAc (20 mM) and varied concentrations (0, 2, 5, 10, 15, 20, 25, 30, 35, 40, 45, 50, and 60 mM) of pyruvate were used. The kinetic parameters of K_m_ and V_max_ were calculated by a nonlinear least-square fitting procedure, using the Michaelis-Menten equation and curve fitting software.

### 4.6. Statistical Analysis

All determinations were performed at least in triplicate and the results were expressed as means ± standard deviations (SD). Statistical analyses were performed using SPSS 17.0 (version 17, SPSS Inc., Chicago, IL, USA). The unpaired Student’s *t*-test was used for the comparison between two groups. Differences were considered to be statistically significant if *p* < 0.05.

## Figures and Tables

**Table 1:** Enzyme characters and kinetic parameters of NAL from different organisms.

Species	Neu5Ac Cleavage				Neu5Ac Synthesis						Reference
	Neu5Ac				ManNAc		Pyruvate				
	K_m_ (mM)	*k_ca_*_t_/K_m_ (s^−1^mM^−1^)	Optimum pH	Optimum Temp (°C)	K_m_ (mM)	*k_ca_*_t_/K_m_ (s^−1^mM^−1^)	K_m_ (mM)	*k_cat_*/K_m_ (s^−1^mM^−1^)	Optimum pH	Optimum Temp (°C)	
Symbiont *Mycoplasma* sp	1.8	3.66	6.0–8.5	70	6.9	1.86	5.9	1.12	7	65	This study
*Corynebacterium glutamicum* ATCC 13032	33.50	0.28	8.4–8.8	40	53.30	0.11	14.70	0.41	8.2–8.4	40	[34]
*Escherichia coli*	3.50	23.75			287.1	0.07	206.1	0.11			[34]
*Escherichia coli*	3.60	23.60	7.7	75					7.7		[30]
*Pasteurella multocida* P1059	4.90	3.27	7.5–8.0		220	0.05	23	0.08	7.5–8.0		[23]
*Clostridium perfringens*	3.20	5.01	7.6	65–70							[42]
*Staphylococcus carnosus* TM300	2	2	7	60–70	149	0.03	14	0.21	7	50	[33]
*Lactobacillus plantarum* WCFS1	1.80	5.60	7.5	70	160	0.03	19.90	0.11	7.5	60	[32]

**Table 2:** Kinetic parameters of MyNal and its various mutants for the cleavage direction.

Enzyme	K_m_ (mM)	*k_cat_* (s^−1^)	*k_cat_*/K_m_ (s^−1^mM^−1^)
Wild-type	1.8 ± 0.2	6.59 ± 0.3	3.66
W189A	1.03 ± 0.3	11.37 ± 0.4	11.04
W189Y	0.5 ± 0.1	12.11 ± 0.3	24.21
W189F	0.36 ± 0.2	12.51 ± 0.1	34.76

## Supplementary Materials

The following are available online at http://www.mdpi.com/1660-3397/16/3/80/s1, Figure S1: SDS-PAGE analysis of the purified MyNal. M: protein marker. Lane 1: cell-free extract without induction. Lane 2: cell-free extract after induction. Lane 3: purified MyNal by Ni^2+^–NTA column. The recombinant MyNal with fusion tags; Figure S2: Alignment of key residues for MyNal and related *N*-acetylneuraminate lyases. The sequences were aligned using ClustalW. The amino acid sequences in the red rectangle indicated sugar-binding domain GXDE of *N*-acetylneuraminate lyases. *Pasteurella multocida* (WP_046339671.1), *Escherichia coli* (WP_033546508.1), *Clostridium perfringens* (Q9S4K9), *Lactobacillus plantarum* (P59407), *Staphylococcus carnosus* (B9DIJ2), *Haemophilus influenza* (P44539), *Fusobacterium varium* (WP_005948757), *Staphylococcus simulans* (WP_002480218), *Clostridium colicanis* (WP_002599534.1), *Aggregatibacter actinomycetemcomitans* (WP_005566544.1), *Haemophilus haemolyticus* (WP_046942498.1), *Actinobacillus suis* (WP_015674266.1), *Gallibacterium genomosp* (WP_039135793.1), *Clostridium butyricum* (WP_058229325.1), *Clostridium hiranonis* (WP_040410204.1), *Leclercia adecarboxylata* (WP_039030055.1), *Peptoclostridium difficile* (WP_044213638.1), *Planomicrobium glaciei* (WP_053167706.1), *Bacillus shacheensis* (WP_059104497.1), *Cetobacterium somerae* (WP_040406735.1), *Atopobacter phocae* (WP_025728679.1), *Staphylococcus aureus* (WP_037589057.1), *Staphylococcus microti* (WP_044359640.1), *Staphylococcus pasteuri* (WP_048803560.1), *Oceanobacillus oncorhynchi* (WP_042529745.1), *Erysipelothrix tonsillarum* (WP_018579629.1), *Lactobacillus salivarius* (WP_003707008.1), *Spiroplasma turonicum* (AKU79951.1), *Hafnia alvei* (WP_004089162.1), and *Salmonella enterica* (WP_023246366.1); Figure S3: HPLC fingerprint spectrum of Neu5Ac. (a) Neu5Ac standard sample. (b) Reaction system; Table S1: Expression level of NAL from different organisms; Table S2: Sequences of the primers used for site-directed mutagenesis. The exchanged nucleotides are shown in bold.
