# Supplementary material for: Molecular Characterization of a Novel N-Acetylneuraminate Lyase from a Deep-Sea Symbiotic Mycoplasma
Source: Mar Drugs. 2018 Mar 5;16(3):80. doi: 10.3390/md16030080 (PMC5867624; doi:10.3390/md16030080)
Supplement: Supplementary file 1 [file marinedrugs-16-00080-s001.docx]

**Supplementary Materials**

**
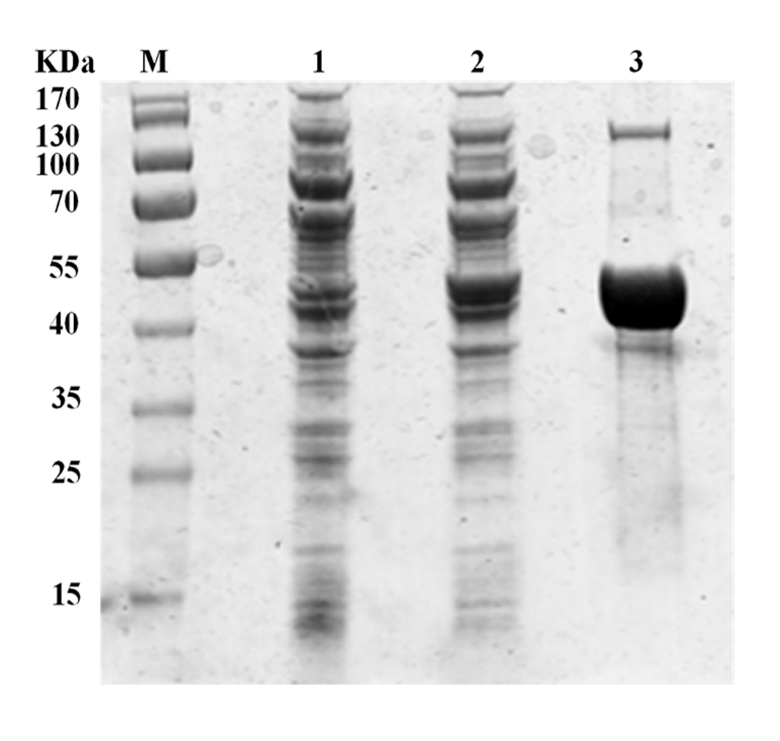
**

**Figure S1.** SDS-PAGE analysis of the purified MyNal. M: protein marker. Lane 1: cell-free extract without induction. Lane 2: cell-free extract after induction. Lane 3: purified MyNal by Ni^2+^–NTA column. The recombinant MyNal with fusion tags.

**
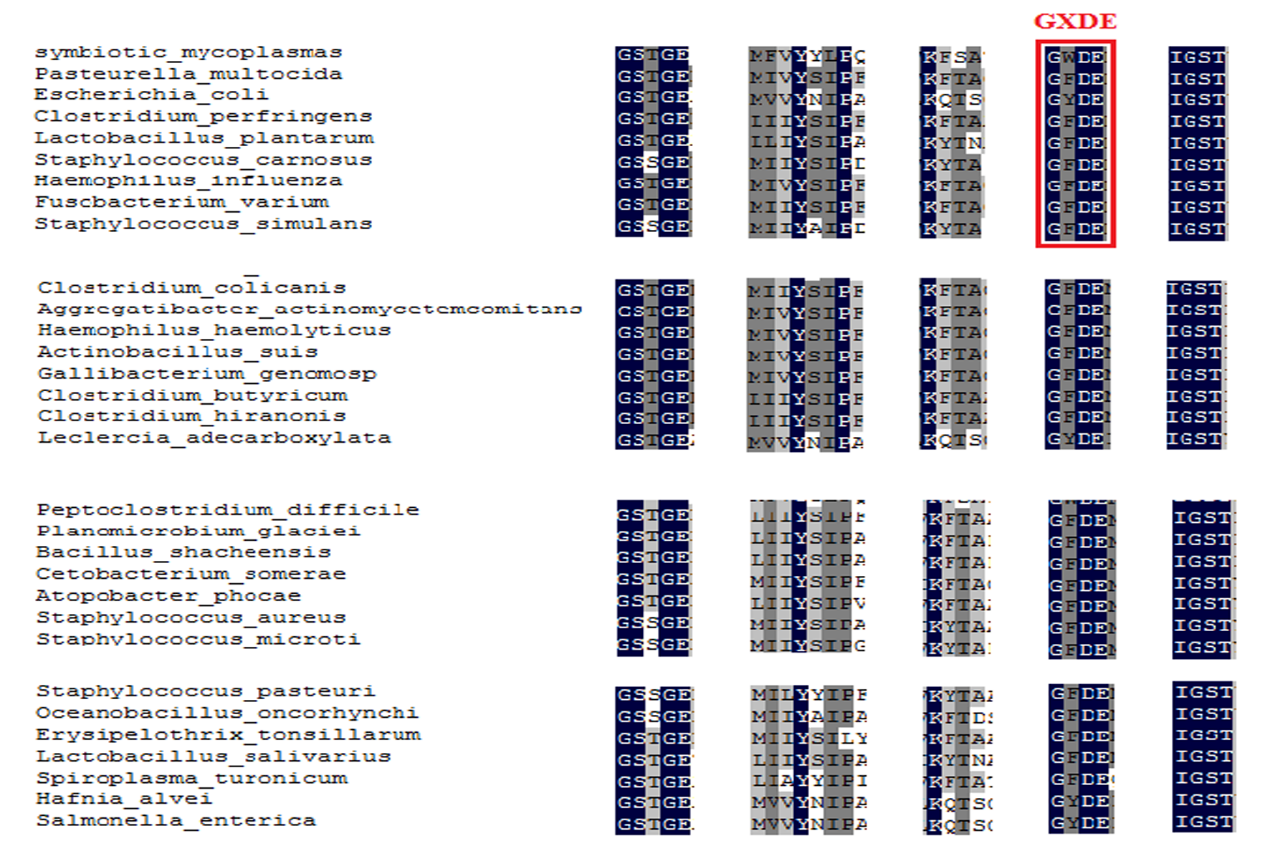
**

**Figure S2.** Alignment of key residues for MyNal and related *N*-acetylneuraminate lyases. The sequences were aligned using ClustalW. The amino acid sequences in the red rectangle indicated sugar-binding domain GXDE of *N*-acetylneuraminate lyases. *Pasteurella multocida* (WP_046339671.1), *Escherichia coli* (WP_033546508.1), *Clostridium perfringens* (Q9S4K9), *Lactobacillus plantarum* (P59407), *Staphylococcus carnosus* (B9DIJ2), *Haemophilus influenza* (P44539), *Fusobacterium varium* (WP_005948757), *Staphylococcus simulans* (WP_002480218), *Clostridium colicanis* (WP_002599534.1), *Aggregatibacter actinomycetemcomitans* (WP_005566544.1), *Haemophilus haemolyticus* (WP_046942498.1), *Actinobacillus suis* (WP_015674266.1), *Gallibacterium genomosp* (WP_039135793.1), *Clostridium butyricum* (WP_058229325.1), *Clostridium hiranonis* (WP_040410204.1), *Leclercia adecarboxylata* (WP_039030055.1), *Peptoclostridium difficile* (WP_044213638.1), *Planomicrobium glaciei* (WP_053167706.1), *Bacillus shacheensis* (WP_059104497.1), *Cetobacterium somerae* (WP_040406735.1), *Atopobacter phocae* (WP_025728679.1), *Staphylococcus aureus* (WP_037589057.1), *Staphylococcus microti* (WP_044359640.1), *Staphylococcus pasteuri* (WP_048803560.1), *Oceanobacillus oncorhynchi* (WP_042529745.1), *Erysipelothrix tonsillarum* (WP_018579629.1), *Lactobacillus salivarius* (WP_003707008.1), *Spiroplasma turonicum* (AKU79951.1), *Hafnia alvei* (WP_004089162.1), and *Salmonella enterica* (WP_023246366.1).

**
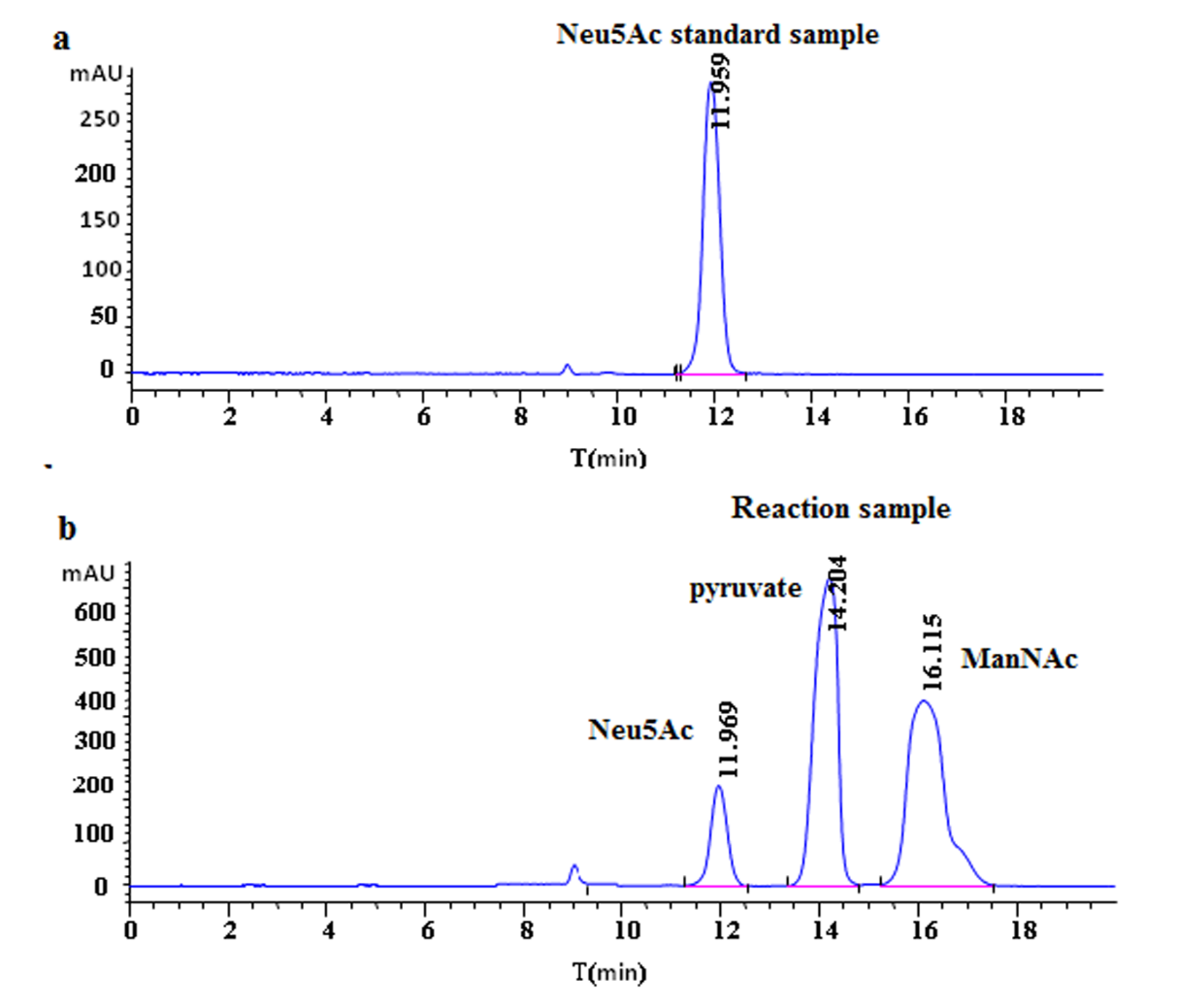
**

**Figure S3.** HPLC fingerprint spectrum of Neu5Ac. (a) Neu5Ac standard sample. (b) Reaction system.

**Table S1** Expression level of NAL from different organisms.

| Species | Concentration (mol/L) | Reference |
| --- | --- | --- |
| Symbiont mycoplasma sp | 9.56 | This study |
| *Corynebacterium*  *glutamicum* ATCC 13032 | 14.55 | 34 |
| *Staphylococcus carnosus* TM300 | 11.51 | 33 |
| *Pasteurella multocida* P1059 | 7.58 | 23 |
| *Lactobacillus plantarum* WCFS1 | 6.51 | 32 |
| *Escherichia coli* K12 | 2.94 | 13 |

**Table S2** Sequences of the primers used for site-directed mutagenesis. The exchanged nucleotides are shown in bold.

| Primer | Primer Sequence |
| --- | --- |
| PET-F | ATGGAAAAATTAACAGGAATTTTTG |
| PET-R | TTATGAAAGATATTTATCAATTATGTT |
| PET-SC-F | TTCTTTATGGATG**G**GATGAAATGCT |
| PET-SC-R | GCATTTCATC**C**CATCCATAAAGA |
| PET-W,Y-F | TTCTTTATGGAT**AT**GATGAAATGC |
| PET-W,Y-R | GCATTTCATC**AT**ATCCATAAAGA |
| PET-W, F-F | TTCTTTATGGAT**TC**GATGAAATGC |
| PET-W, F-R | GCATTTCATC**GA**ATCCATAAAGA |
| PET-W, A-F | TTCTTTATGGA**GC**GGATGAAATGC |
| PET-W, A-R | GCATTTCATCC**GC**TCCATAAAGA |
